# Supplementary figures and images for: Screening and diversity analysis of Dibutyl phthalate degrading bacteria in agricultural soil in Chengdu, China
Source: PLoS One. 2024 Dec 18;19(12):e0310979. doi: 10.1371/journal.pone.0310979 (PMC11654953; doi:10.1371/journal.pone.0310979)

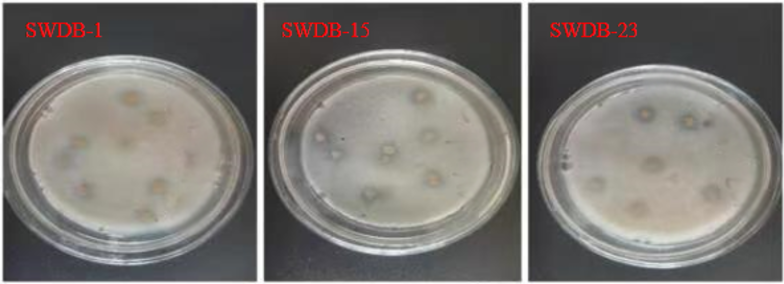

Supplement: S1 Fig — (TIF) [file pone.0310979.s002.tif]

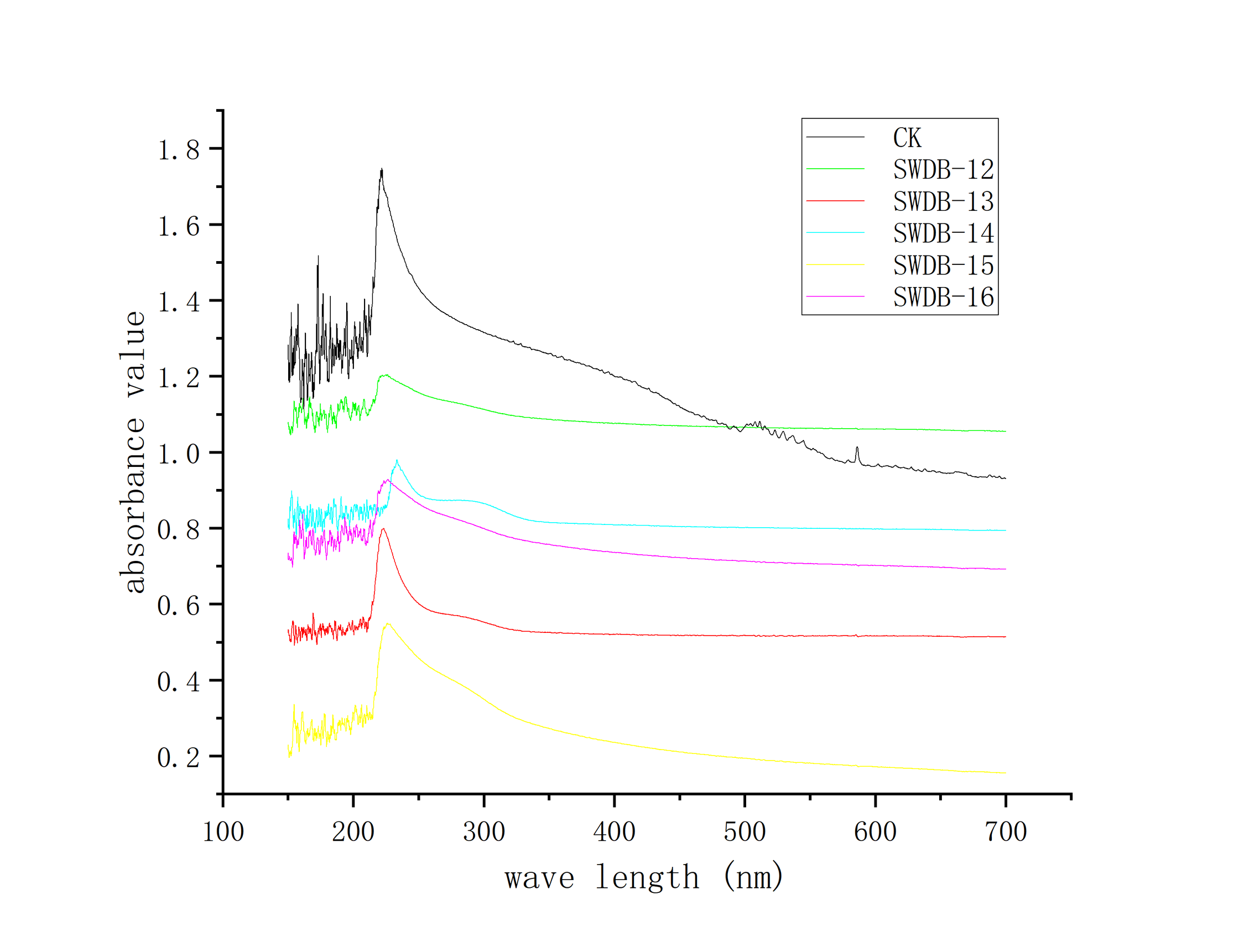

Supplement: S2 Fig — (ZIP) [file pone.0310979.s003.zip › Supporting Fig 2. SWDB12-16.tif]

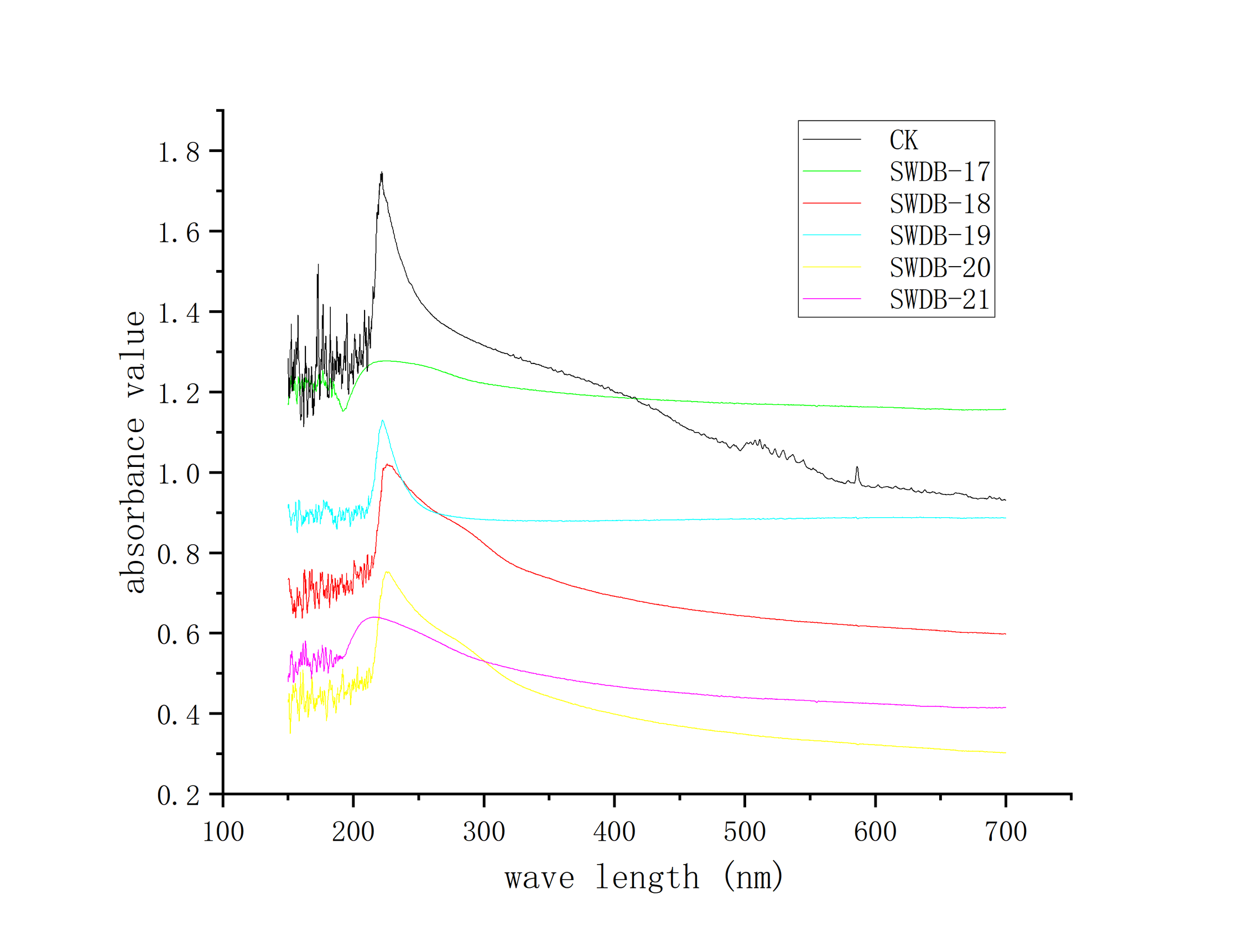

Supplement: S2 Fig — (ZIP) [file pone.0310979.s003.zip › Supporting Fig 2. SWDB17-21.tif]

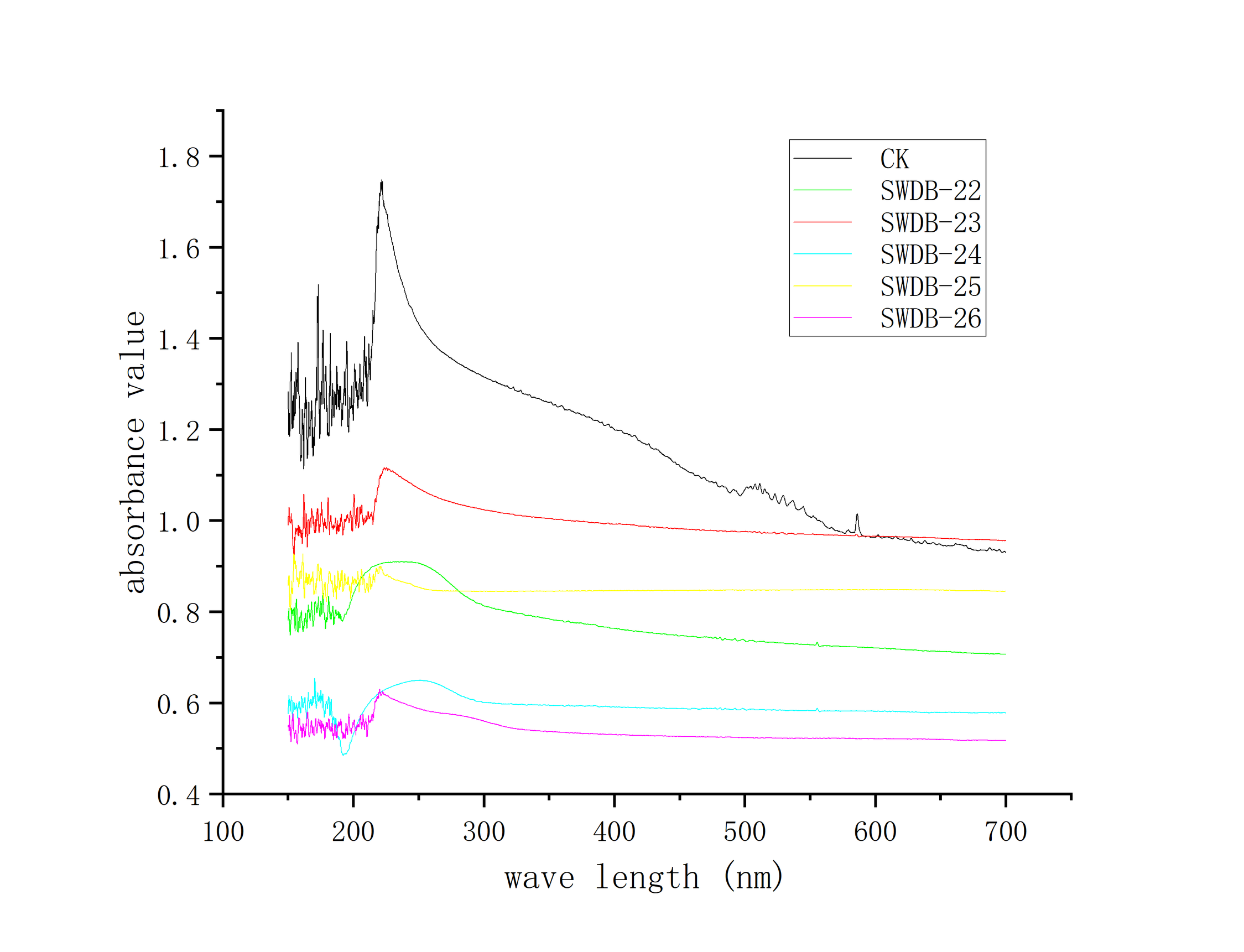

Supplement: S2 Fig — (ZIP) [file pone.0310979.s003.zip › Supporting Fig 2. SWDB22-26.tif]

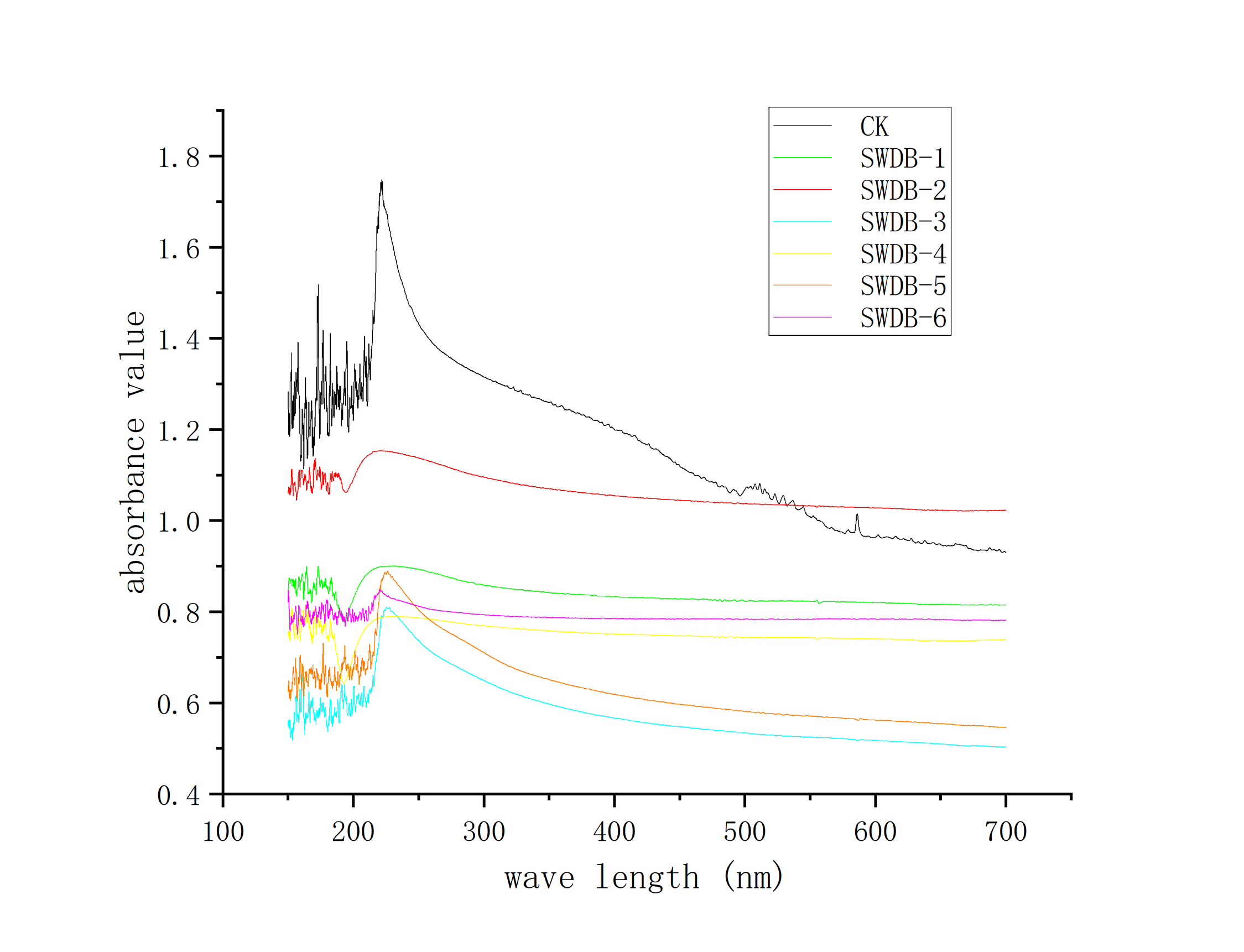

Supplement: S2 Fig — (ZIP) [file pone.0310979.s003.zip › Supporting Fig 2. SWDB1-6.tif]

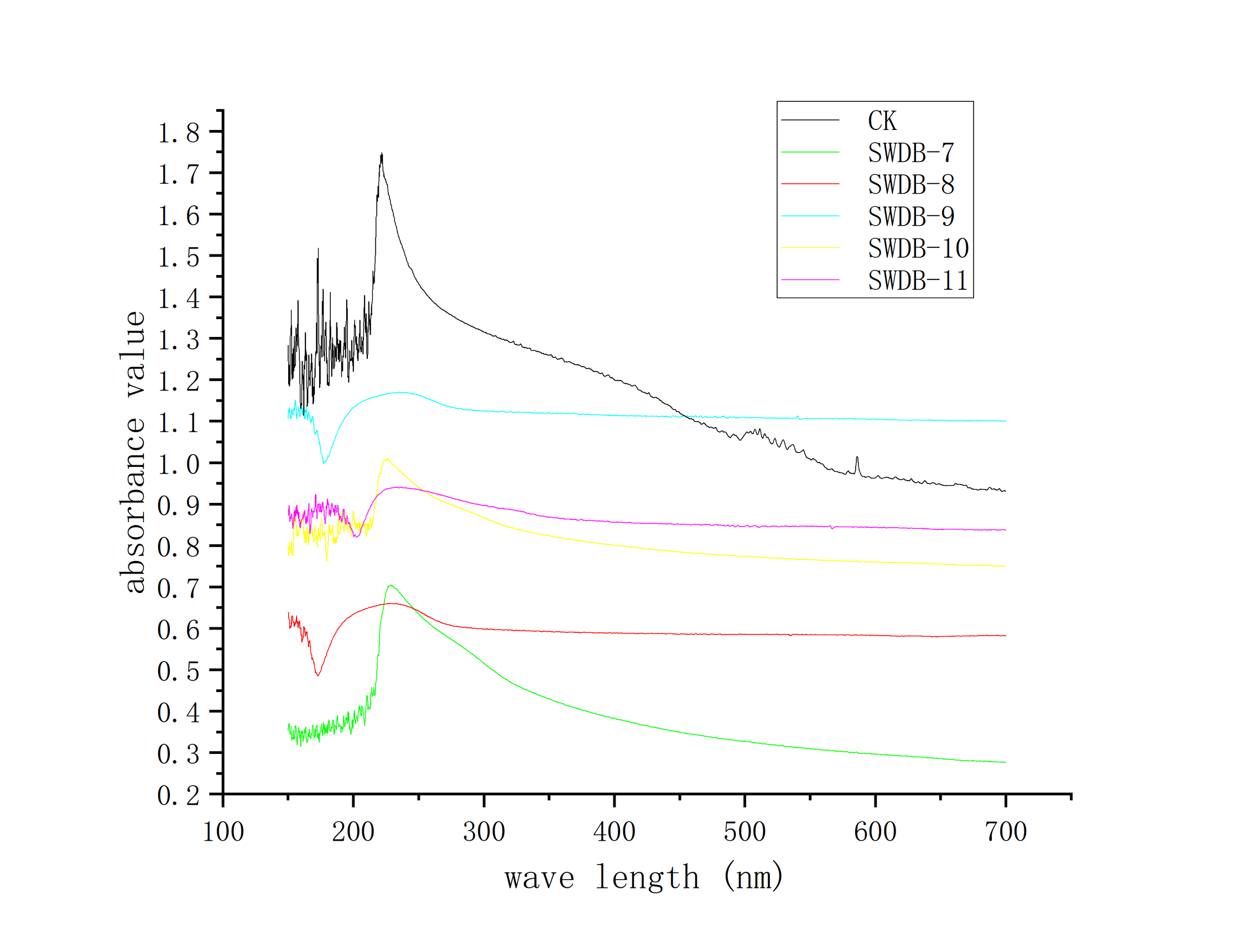

Supplement: S2 Fig — (ZIP) [file pone.0310979.s003.zip › Supporting Fig 2. SWDB7-11.tif]
