# Supplementary material for: Screening and diversity analysis of Dibutyl phthalate degrading bacteria in agricultural soil in Chengdu, China
Source: PLoS One. 2024 Dec 18;19(12):e0310979. doi: 10.1371/journal.pone.0310979 (PMC11654953; doi:10.1371/journal.pone.0310979)
Supplement: Attachment — Submitted filename: Response to Reviewers.docx [file pone.0310979.s004.docx]

Comments and Suggestions for Journal Requirements:

1. Please ensure that your manuscript meets PLOS ONE's style requirements, including those for file naming.

Reply: We have revised the manuscript and file naming according to PLOS ONE's style requirements.

2. In your Methods section, please provide additional information regarding the permits you obtained for the work. Please ensure you have included the full name of the authority that approved the field site access and, if no permits were required, a brief statement explaining why.

Reply: No permits were required for the sampling site. For details, see the attachment naming "a brief statement on the permits of the sampling site".

Comments and Suggestions for Editor:

1. The chemicals and materials need to be supplemented with a grade to indicate that they have the appropriate purity for research.

Reply: Accepted and revised other chemical reagents’ grade in “Materials and Methods” section.

2. Please present the QA/QC method to demonstrate the necessary parameters of the DBP analytical method, such as recovery efficiency, repeatability, and LOQ.

Reply: Accepted and presented the relevant content in “Materials and Methods” section.

3. The manuscript should illustrate more results using charts. For example, in section 3.3.1, a UV-Vis spectrum is required. Graphs showing the degradation of DBP over time should also be presented in chart form. Additionally, it may be necessary to include HPLC spectra to show the degradation before and after the degradation experiment.

Reply: The images of ultraviolet and visible spectrophotometry spectrum have been supplemented. However, wing to the malfunction of the computer that stored figures for results of HPLC chromatograms in our laboratory, the relevant data has been lost. Currently, we are unable to provide the relevant figures and we sincerely apologize for that.

Comments and Suggestions for Reviewer #1:

1- Write the name of the country China for Dayi County. Also, for all cities in the abstract.

Reply: Accepted and it is revised.

2- The following related articles should be cited in the introduction.

Reply: Accepted and it is revised. However, this article (https://doi.org/10.1016/j.scitotenv.2019.02.385) has already been cited as reference [20] in the introduction.

3- Novelty over old published articles should be highlighted in the abstract.

Reply: Accepted and it is revised in “Introduction” section .

4- Line 64- 66 add reference

Reply: Accepted and it is revised.

5- Line 132, add country name. also, in line 136

Reply: Accepted and it is revised.

6- Add reference for the method in line 153

Reply: Accepted and it is revised.

7- Line 237, write full name for R2

Reply: Accepted and it has been improved.

8- Line 252, remove and

Reply: Accepted and it is removed.

9- Line 269, as shown in Table 3-1.; it should be Table 1.also, in 271

Reply: Accepted and it is revised.

10- Remove repeated information from line 350- 361

Reply: Accepted and it is removed.

11- Study limitation and future plan should be suggested

Reply: Accepted and it has been improved.

Reviewer #2:

1) Line 52, it is not usual that the phrase “in our nation” be used in scientific literature. Please replace it with the country name.

Reply: Accepted and it has been changed into “in China”.

2) Line 75, instead of using the word “cheap”, you can say “cost-effective” or “relatively cheaper”

Reply: Accepted and it is corrected.

3) Line 111, replace on with in

Reply: Accepted and it is corrected.

4) Lines 126-132 are better suited at the beginning not towards the end of the introduction.

Reply: Accepted and it is revised.

5) Line 135, no mention of the number of samples collected? These should be clarified

The number of samples collected was not mentioned in the previous manuscript. It has been improved and revised in the "Materials and Methods" section.

6) Line 137, should state that these are coordinates and not just numbers.

Reply: Accepted and it is revised.

7) Line 149, the statement seems to be unrelated to text before it and should be integrated into it properly.

Reply: Accepted and it is revised.

8) Line 154, same as above and additionally the first sentence is not clear here and the second sentence is in brackets which makes no sense for a sentence!

Reply: Accepted and it is revised.

9) Line 158, it is better to use the word “Each” instead of “The” at the start of the sentence.

Reply: Accepted and it is revised.

10) Line 168, “The bacterial suspension should be” I believe that it was already and thus this sentence should be re-written.

Reply: Accepted and this sentence has been re-written.

11) Line 172, the entire sentence needs to be re-written.

Reply: Accepted and the entire sentence has been re-written.

12) Line 177, the sentence “After that, inoculate the pure…..” needs to be re-written correctly.

Reply: Accepted and the sentence has been re-written.

13) Line 218, “then use sterile water….” should be re-written correctly.

Reply: Accepted and this sentence has been re-written.

14) Line 228, “place the conical…..” should be re-written correctly.

Reply: Accepted and this sentence has been re-written.

15) Line 236, what do the authors mean by “external standard method”? also, I would say “a standard curve was constructed” instead of construct a standard curve.

External standard method is one of the common methods of instrument analysis, and it is a kind of comparative method. First, a certain amount of standard product is added in a blank solvent according to the gradient to make a reference sample. Then, measuring the peak areas corresponding to the different reference samples. After that, based on the resulting peak area and the corresponding sample concentration, plot the standard curve. Finally, measuring the peak area of the sample with unknown concentration and combining the resulting standard curve calculating the concentration of the sample with unknown concentration.

About “construct a standard curve”, it is corrected, changing into “a standard curve was constructed”.

16) Line 242, the second sentence needs to be re-written.

Reply: Accepted and this sentence has been re-written.

17) Line 304, “Then the degradation efficiency can be calculated by comparing it to the DBP standard curve” you mean it can or it was?

Yes, it can. The specific calculation method and process are detailed in the interpretation of the "external standard method".

18) Table 3-2: the letters should be put as superscripts instead of being next to the numbers.

Reply: Accepted and it is revised.

19) In line 308, But the table doesn't exactly show which ones were in the bacterial fermentation broth? This distinction needs to be made clear in the table itself.

The OD_600_, Residual DBP concentration, Degradation efficiency and absorbance difference at maximum absorption wavelength were in the bacterial fermentation broth. It has been refined and modified in the manuscript.

20) Line 345, replace the comma with a full stop.

Reply: Accepted and it is corrected.

21) Line 446, “the” should be capitalized correctly.

Reply: Accepted and it is corrected.

22) In addition, the statistical model used for the data analysis was not mentioned nor was the software used for the analysis which should typically be in the materials and methods section.

Reply: Add “statistical analysis” in the “materials and methods” section.

Reviewer #3:

1.It will be interesting to know what the degraded components of Dibutyl Phthalate were using the bacteria isolated from this study. The bacteria could have degraded the Dibutyl Phthalate to other substance that can be regarded as pollutants.

In previous manuscript’ "Discussion" section, relevant content regarding the degradation mechanism of DBP degrading bacteria has already been presented. In future experiments, we will conduct further research on the degradation mechanism of DBP degrading bacteria which we had separated and gain an understanding of the degradation products of each DBP degrading bacterium.

2.The author made use of unclear (ambiguous) statements such as the ''using the pure culture approach'', ''purified by the dilution coating plate method and quadrant streaking method''. The experimental design of this research make some conclusions that can not to be affirmative. For one of the bacteria strains the author suggests it to be a new species based on 16S RNA blast only. This alone can not make a bacteria to be a new species. The author thinks a particular genera is abundant but based on the isolation and characterization technique used the abundance of that genera might not be the true picture.

About unclear (ambiguous) statements and inconclusive conclusions, we have revised the article according to the Reviewer’ comment. About SWDB-7, we only thought that it is a potential new species. In future studies, the polyphasic taxonomy of SWDB-7 would be carried out in future studies to identify which species SWDB-7 belongs and whether SWDB-7 is a potential new species. And about prominent genus, we have revised the article according to the Reviewer’ comment.

3.Why did the author selected some colonies on the basal medium and not all. "Colonies with clear morphology and good growth but without transparent circle were separated and purified." I would have thought ability of the bacteria to grow on the DPB would have made them a presumptive bacteria capable of degrading DPB.

Since we hold the belief that the bacteria in colonies with clear morphology or good growth but without transparent circle possess a superior degradation ability on minimal salt solid medium. Then, use ultraviolet and visible spectrophotometry and HPLC to validate their degradation capability.

4.Not sure the need of purification in line 135

Reply: Accepted and it is removed.

5.The soil could have been used without air drying not sure the use of air drying line 140

Reply: Accepted and sampling description had been revised.

6.The unit should be rpm but rmp was used in the manuscript

Reply: Accepted and it is corrected.

7.10-1 bacterial suspension in line 167 could have 10 fold serial dilution

Reply: Accepted and it is revised.

8.Not sure if a single gene can be assembled into contigs line 199

Reply: A single gene can’t be assembled into contigs. The relative description has been revised.

9.The isolated colonies in line 214 does not suggest each isolated bacteria

Reply: Accepted and the relative description has been revised.

10.Both rpm and g was used in line 217. I suggest that a particular unit be used either g or rpm

Reply: Accepted and it is corrected.

11.I am confused with the word thallus in line 219

Reply: The relative description has been revised.

12.Was the UV reading at 200-350 a good wavelength for measurement of the breakdown of DPB

Yes, it was. By reading the relevant literature and materials, such as: Lu MY. (2019) Isolation of PAEs-degrading strain、analysis of its degradation pathway, cloning of hydrolase gene and characterization of its encodinig enzyme. M.Sc. Thesis, *Nanjing Agricultural University*. Xia FY. (2002) Biodegradability Research of Phthalic Acid Esters. D.Eng. Thesis, *Zhejiang University*. and so on, we found that the scanning range of 200-350 nm can contain the measurement wavelength required for DBP.

13.In the results section line 264 Table 2 was cited before table 1. The description of table 2 in line 261-264 is not what we have in the exact Table 2.

Reply: Accepted and it is revised.

14.Table 3-1 might be moved to a supplementary file. Instead of having this table, it can be presented in a summary of presenting the gene length of the 16S RNA what range it is how many of the strains falls into what genus and a similarity range for the blast. A summary is ok and the table can then be moved into supplementary file.

Reply: Accepted and it is revised.

Reviewer #4:

1. It would be beneficial to include a brief overview of the current state of DBP pollution in China and globally to provide a broader context.

Reply: Accepted and it is revised in “Introduction” section.

2. It would be helpful to include more details on the soil sampling process, such as the depth of sampling and the number of replicates, to ensure that the study's robustness is clear.

Reply: Accepted and it is revised in “Materials and Methods” section.

3. The use of 16S rDNA sequencing for taxonomic identification is appropriate, but the manuscript could benefit from a discussion on the limitations of this method and any additional techniques that could be used to confirm the identity of the potentially new species SWDB-7.

Reply: Accepted and it is revised in “Discussion” section.

4. The manuscript could be strengthened by discussing the implications of the findings for practical applications, such as the potential for using these bacteria in field-scale bioremediation projects.

Reply: Accepted and it is revised in “Discussion” section.

5. It could be strengthened by discussing the implications of these findings for future research or practical applications in bioremediation.

Reply: Accepted and it is revised in “Discussion” section.

6. The abstract mentions “vegetable plots contaminating plastic mulch” which could be rephrased for clarity to “vegetable plots contaminated with plastic mulch”.

Reply: Accepted and it is corrected.

Reviewer #5:

1. The title needs to be clarified.

Reply: Accepted and the title has been improved.

2. I highly recommend reviewing the grammar of the paper.

Reply: Accepted and the grammar of the paper has been revised.

3. Introduction section;

1)- Line 54, change country exceeds to “countries exceed”.

Reply: Accepted and it is corrected.

2)- Line 56, write “Based”

Reply: Accepted and it is corrected.

3)- line 60, 77, 80, and 99, Change to agricultural soil.

Reply: Accepted and it is corrected.

4)- Line 66, remove “And”.

Reply: Accepted and it is removed.

5)- Line 128, remove “so as”.

Reply: Accepted and it is removed.

4. Results section;

1)- Line 230, what is the meaning of 1.75?

Reply: 1.75 means that the average absorbance corresponding to the maximum absorption wavelength of the bacterial fermentation broth produced by the control. The relative description has been revised.

2)- The image of transparent rings on culture plates.

Reply: We have added the image of transparent rings on culture plates as S1 Fig. in “Supporting information” section.
